# Intensive neurorehabilitation for patients with prolonged disorders of consciousness: protocol of a mixed-methods study focusing on outcomes, ethics and impact

**DOI:** 10.1186/s12883-021-02158-z

**Published:** 2021-03-22

**Authors:** Manju Sharma-Virk, Willemijn S. van Erp, Jan C. M. Lavrijsen, Raymond T. C. M. Koopmans

**Affiliations:** 1 Radboud Institute for Health Sciences; Department of Primary and Community Care, Radboud University Medical Centre, Nijmegen, The Netherlands; 2 PZC Dordrecht, Dordrecht, The Netherlands; 3 Accolade Zorg, Bosch en Duin, The Netherlands; 4 Libra Revalidatie & Audiologie, Tilburg, The Netherlands; 5 Joachim en Anna, Centre for Specialized Geriatric Care, Nijmegen, The Netherlands

**Keywords:** Prolonged disorders of consciousness, Acute brain injury, Rehabilitation outcomes, End-of-life decisions

## Abstract

**Background:**

Prolonged disorders of consciousness (PDOC) are amongst the severest sequelae of acquired brain injury. Evidence regarding epidemiology and rehabilitation outcomes is scarce. These knowledge gaps and psychological distress in families of PDOC patients may complicate clinical decision-making. The complex PDOC care and associated moral dilemmas result in high workload in healthcare professionals.

Since 2019, all PDOC patients in the Netherlands have access to intensive neurorehabilitation up to 2 years post-injury provided by one rehabilitation center and four specialized nursing homes. Systematic monitoring of quantitative rehabilitation data within this novel chain of care is done in a study called DOCTOR. The optimization of tailored PDOC care, however, demands a better understanding of the impact of PDOC on patients, their families and healthcare professionals and their views on rehabilitation outcomes, end-of-life decisions and quality of dying. The **T**rue **O**utcomes of **PDOC** (TOPDOC) study aims to gain insight in the qualitative outcomes of PDOC rehabilitation and impact of PDOC on patients, their families and healthcare professionals.

**Methods:**

Nationwide multicenter prospective cohort study in the settings of early and prolonged intensive neurorehabilitation with a two-year follow-up period, involving three study populations: PDOC patients > 16 years, patients’ family members and healthcare professionals involved in PDOC care. Families’ and healthcare professionals’ views on quality of rehabilitation outcomes, end-of-life decisions and dying will be qualitatively assessed using comprehensive questionnaires and in-depth interviews. Ethical dilemmas will be explored by studying moral deliberations. The impact of providing care to PDOC patients on healthcare professionals will be studied in focus groups.

**Discussion:**

To our knowledge, this is the first nationwide study exploring quality of outcomes, end-of-life decisions and dying in PDOC patients and the impact of PDOC in a novel chain of care spanning the first 24 months post-injury in specialized rehabilitation and nursing home settings.

Newly acquired knowledge in TOPDOC concerning quality of outcomes in PDOC rehabilitation, ethical aspects and the impact of PDOC will enrich quantitative epidemiological knowledge and outcomes arising from DOCTOR. Together, these projects will contribute to the optimization of centralized PDOC care providing support to PDOC patients, families and healthcare professionals.

## Background

Thanks to the advances in emergency and intensive care medicine in the last decennia, survival of patients with acquired brain injury has increased. The reverse side of the coin is a rise in severe chronic conditions like prolonged disorders of consciousness (PDOC) [1, 2]. PDOC is an umbrella term used for rare conditions including unresponsive wakefulness syndrome (UWS) and minimally conscious state (MCS), where patients respectively show no or only minimal and inconsistent evidence of awareness of themselves and their environment [2–5]. These conditions may be transient, as some patients may recover from UWS to MCS and eventually emerge into a fully conscious state [2]. Others, however, remain with chronic PDOC for the rest of their lives [2, 5]. With appropriate treatment and specialized rehabilitation, recovery of consciousness has been reported in two-third of patients with PDOC following traumatic brain injury [6, 7]. One-fifth of MCS patients may regain functional independence, whereas nearly 18% demonstrated employment potential [6, 8]. Although reports of late recovery are available, the first 2 years post-injury are particularly crucial: this is the phase when patients are most likely to recover but are also most vulnerable to life-threatening complications [2, 9, 10].

### Challenges in PDOC-care

Providing tailored care to PDOC patients and their families is a complex task due to several diagnostic, prognostic, therapeutic and ethical challenges [2, 5].

First of all, PDOC represent a dramatic condition in itself with a sudden and huge impact on both patients and their families. PDOC are generally considered to have a poor outcome [11–15]. UWS has even been described as ‘a fate worse than death’ [16]. PDOC confronts families with complex intertwined feelings of grief, hope and ambiguous loss at the same time [17, 18]. Secondly, a low worldwide prevalence for UWS of 0.2 to 6.1 per 100.000 individuals of the total population makes it difficult for clinicians to master the required diagnostic and therapeutic expertise [2, 11, 19]. Third, there is a consistent high misdiagnosis rate of ca. 40% in PDOC [19–21]. This may result in inefficient pain management, therapeutic nihilism and inappropriate treatment decisions [19, 20, 22]. Fourth, even with the right diagnosis, there are no reliable outcome predictors in PDOC [2, 23]. This makes it impossible for clinicians to predict which patients are most likely to benefit from specialized rehabilitation programmes and achieve a meaningful recovery [2, 5, 24]. This is particularly challenging in early stages of PDOC, as significant neurological and functional improvement may become apparent up to 2 years post-injury [2, 7, 25]. Fifth, although patients can benefit from early rehabilitative interventions, access to specialized rehabilitation is often limited, even in developed countries [11, 26]. Sixth, treatment decisions in PDOC, especially in the absence of advance directives, are challenging [5, 27]. These decisions frequently involve complex moral dilemmas and may sometimes even result in conflicts between family members and healthcare professionals [28–30]. End-of-life decisions (EOLD) are inevitable in the course of PDOC care. However, physicians cannot rely on scientific evidence regarding prognosis and outcomes in PDOC [2, 5]. The prognosis of PDOC becomes clear with the course of time. While further treatment may no longer result in the improvement of consciousness and level of functioning, it may become increasingly difficult for patients’ families to let them go [31, 32].

All these challenges may result in suboptimal care for PDOC patients and their families [11]. Consequently, these patients and their families not only face tragic consequences of PDOC itself but are also confronted with logistic issues and ‘disordered’ PDOC care [33].

### End-of-life decisions, ethical dilemmas and dying in PDOC

Besides the aforementioned issues in a recovery-targeted treatment, controversies regarding EOLD and the process of dying in PDOC attract frequent worldwide media attention [34–37]. The views on (dis) continuation of life sustaining treatment (LST) vary widely in different cultural and geographical settings [12]. In the Netherlands, physicians are allowed to withdraw LST in the absence of chances of recovery of consciousness [38–40]. Most data on the process of EOLD and dying of UWS patients comes from studies in chronic care settings [31]. In the early 2000’s nine out of 43 UWS patients in Dutch nursing homes died after a physician’s decision to withdraw clinically assisted nutrition and hydration (CANH), whereas 24 died after a decision not to treat a new complication [41]. A recent Dutch cohort on UWS patients reported a marked increase in deaths following a physicians’ decision to withdraw CANH [11].

Generally, dying after withdrawal of CANH in these patients is described as a peaceful process [31, 42, 43]. However, some people may find it burdensome or even “appalling” to watch deterioration of the physical appearance of their loved one after withdrawal of CANH and may also fear for the symptoms of pain and discomfort [43–45]. Little is known about EOLD and dying in acute care and rehabilitation settings. How and when during the rehabilitation process, treatment decisions are changed into EOLD and the factors influencing these decisions have not been studied before, particularly in MCS patients.

### Quality of outcomes and family aspects

Families of PDOC patients often have to balance between providing care to their loved one, being their spokesperson and managing their own emotional, psychological, social and financial struggles [17, 18, 46, 47]. A recent systematic review reported the presence of depression in 33–70% of family members of PDOC patients in various clinical settings whereas prolonged grief disorder was present in 15–60% and did not decrease over time [18, 47].

Data on the quality of life in PDOC patients after intensive neurorehabilitation is scarce. The views of PDOC patients and their families regarding their expectations and their views on the quality of achieved rehabilitation outcomes have not been studied extensively. A question that family members of PDOC patients may therefore ask when it comes to the (quality of) outcomes of intensive neurorehabilitation programmes is: “Is it worth it?”

### Impact of providing care on healthcare professionals

Healthcare professionals struggle finding a balance between providing complex and intensive PDOC care, which is often sub-optimally financed or facilitated, while keeping up with the demands of the families of PDOC patients [48]. The burnout rates in healthcare professionals are high [28, 48, 49]. In a study of 523 healthcare professionals, one out of five members of the medical staff working with PDOC patients in both rehabilitation and nursing-home settings reported presence of burn-out symptoms [48]. The burn-out rate was found to be higher in the nursing staff than in physicians and higher (23 vs 14%) in nurses working in nursing homes as compared with those working in rehabilitation settings [48]. Only one study analyzed the impact of working with PDOC patients on healthcare professionals working in a hyperacute rehabilitation setting (on an average of 6 months post-injury), who described their work as both rewarding and challenging at the same time [50]. Rewarding experience was associated with seeing change in patients’ condition, supporting families, the feelings of satisfaction and pride in working with a qualified team. Dealing with death of patients and demands or distress of families was characterized as a negative aspect of providing PDOC care [50]. However, only 24% of the study participants were physicians and nurses. As the chances of recovery become slimmer further down the rehabilitation trajectory, the needs of healthcare professionals may also change, leaving them with different or higher burdens. Moreover, healthcare professionals working in different settings, like rehabilitation centers or nursing homes, may experience different challenges and may have distinct needs. The factors associated with work-related stress, experiences and needs of healthcare professionals involved in PDOC care and a comparison between aspects of providing care in these different care settings has not been studied before.

Summing up, multiple challenges and knowledge gaps exist in PDOC care regarding epidemiology, quality of rehabilitation outcomes, EOLD, dying process and the impact of PDOC on families and healthcare professionals. Further research is crucial not only to help improve the quality of provided care to this vulnerable group of patients but also to support their families and health care professionals.

Recent developments in the Netherlands provide a unique opportunity to optimize PDOC care and address aforementioned aspects. Since 2019, all PDOC patients, irrespective of their age have access to specialized intensive neurorehabilitation programme. In April 2019, a systematic scientific quantitative data registry called DOCTOR (early intensive neurorehabilitation in patients with prolonged **D**isorders **O**f **C**onsciousness- **T**reatment and **O**utcome **R**egistry) was initiated, in order to prospectively monitor all PDOC patients admitted to intensive neurorehabilitation programme [51]. DOCTOR will quantitatively address research questions about epidemiological aspects of PDOC and rehabilitation outcomes. The current **T**rue **O**utcomes of **PDOC** (TOPDOC) study will enhance and enrich the quantitative outcomes of DOCTOR using qualitative approach and address what it means for the patients, families and healthcare professionals to be confronted with PDOC.

## Objective

TOPDOC aims to provide insight in the process of clinical decision-making, ethical dilemmas, quality of outcomes and dying in PDOC patients undergoing intensive neurorehabilitation during first 2 years post-injury and understanding the impact of providing care to PDOC patients on their families and healthcare professionals.

TOPDOC will address following research questions:

1. Which EOLD are made in PDOC during first 2 years post-injury and why?
2. Which ethical dilemmas occur during this period?
3. What is the quality of outcomes in PDOC patients from their own perspective and or that of their families?
4. How do PDOC patients die and what are the perceptions and views of their families, physicians and nurses involved in PDOC care regarding the quality of dying?
5. What is the impact of providing care to PDOC patients on the health care professionals? Which barriers and facilitators can be identified?

## Methods

### Study design

The TOPDOC study is a multicenter, longitudinal prospective cohort study with a total duration of 4 years. In this mixed-methods study, both quantitative and qualitative data will be used.

### Setting

**Fig. 1:**
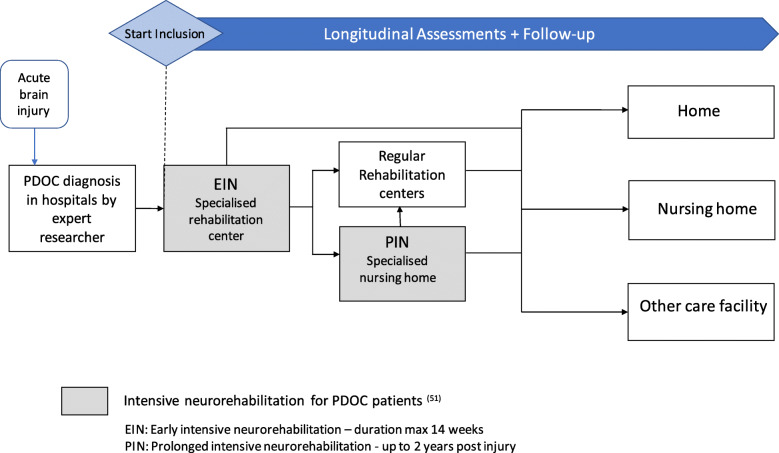
Intensive neurorehabilitation for PDOC patients in the Netherlands [51]

Patients will be included during the first 2 years of the study, starting with their enrollment in the early intensive neurorehabilitation (EIN) programme, provided by a single rehabilitation center. Last patient will be included around the end of the second year [51]. All patients will have a follow-up of 2 years, throughout the PDOC chain of care, including one of the four nursing homes providing prolonged intensive neurorehabilitation (PIN) and subsequent transfer to other care facilities or home. See Fig. 1.

After in-hospital diagnostic assessment by one of the academic experts of “EENnacoma” (= one after coma) network (an academic network of expertise for post-acute and long-term care for patients with severe acquired brain injury), patients diagnosed with PDOC are transferred to EIN for up to 14 weeks [51] (https://www.eennacoma.net/). Patients who do not regain consciousness during EIN are eligible for PIN up until a maximum of 24 months post-injury in one of the four specialized nursing homes. Besides intensive medical care, EIN and PIN focus on prevention and management of (long-term) complications, repeated assessments of level of consciousness, multidisciplinary therapy to improve consciousness and functional level and providing support and council for patients’ families [51]. EIN involves 25 therapy sessions a week and is coordinated by physiatrists, while elderly care physicians (ECP), previously known as nursing home physicians, are in charge of PIN, which consists of 10 therapy sessions a week. ECPs are particularly specialized in the management of severe chronic conditions, shared decision-making and end-of-life care [52]. At any point during EIN or PIN, patients may be discharged, depending on their neurologic and functional outcomes, to either regular rehabilitation centers, other care facilities like long-stay nursing home, hospice or home.

### Study population

Three study populations are included: 1. Population A: PDOC patients > 16 years participating in the EIN program (a subgroup of the DOCTOR study population) [51], 2. Population B: family members (i.e., primary representatives) of population A, 3. Population C: healthcare professionals involved in PDOC care. Inclusion and exclusion criteria are presented in Table 1.

**Table 1:** Inclusion and exclusion criteria of the three study populations

	Population A	Population B	Population C ^**b**^
**Participants**	Patients with PDOC admitted to the EIN program	Family members of population A	• Treating physicians • Nurses • Multidisciplinary team^c^
**Inclusion criteria**	• Age > 16 years • Diagnosis of PDOC based on CRS-R^a^ assessment by experienced researcher • Medically stable, as judged by treating physician	• Age > 16 years • Primary legal representative of patients included in population A	Currently working or was working until a maximum of 12 months ago, with PDOC patients within the Dutch chain of PDOC care
**Exclusion criteria**	• Presence of progressive brain injury including neurodegenerative disorders • Uncontrollable epilepsy	Non-fluency in Dutch or English	Non-fluency in Dutch or English

^a^*CRS-R* Coma recovery scale revised: Standardized assessment tool for differentiating levels of consciousness by observation of pa tients’ response to different stimuli [52]

^b^Population C involves different participants per study theme- (see details in study methodology)

^c^Multidisciplinary team includes besides physician and nurses other disciplines actively involved in PDOC rehabilitation like physiotherapist, speech therapist, occupational therapist, social worker and psychologist

All patients and their family members participating in the EIN program who consented for DOCTOR are also eligible for TOPDOC. Based on available 14 beds for EIN and the estimated number of PDOC patients aged 16 or above who meet the inclusion criteria, we expect to include at least 72 patients in the total study course of 4 years.

### Characteristics of study population

For all three study populations, demographic features like age, gender, education level/occupation will be registered. For population A, the following variables will be characterized: living situation before brain injury, diagnosis, brain injury type, date of brain injury, days post-injury at start of the study, medication use in the last 3 weeks prior to death, signs of discomfort or pain during terminal phase, discharge status and mortality data. For population B, relation to the patient will be reported. For population C, besides occupation and education-level, years of working experience with PDOC patients will also be registered.

### Study methodology per theme and data collection

#### Treatment and EOL-decisions in PDOC

From the medical records, information will be retrieved regarding available written advance directives of the patients and pre-decided treatment decisions e.g., cardiopulmonary resuscitation in case of cardiac arrest. Physicians involved in the process of clinical decision-making during first 2 years post-injury will be approached to fill in a comprehensive questionnaire regarding: (1) (non) treatment decisions (2) intended treatment goals and (3) expected outcomes. The quantitative data acquisition will involve all patients included in TOPDOC.

Physicians will be requested to participate in semi-structured qualitative interviews regarding their perspectives on the decision-making process during PDOC. These interviews will primarily focus on how and when EOLD come to place during the first 2 years post-injury. Factors involved in EOLD, role of families in this process and discrepancies experienced by physicians, if any, will be explored.

Interviews will be held on the basis of purposeful sampling in a minimum of 6 cases with a peculiar decision-making process. Interviews will be performed at 3, 6, 12 and 24-months post-injury. These timepoints are chosen as they are prognostically important in different PDOC etiologies (traumatic vs non-traumatic) and thought to be related to the window of opportunity for evaluation of treatment-goals and EOLD [2, 5].

#### Ethical dilemmas in PDOC

Ethical dilemmas occurring in first 2 years post-injury will be studied using multidisciplinary moral deliberations (MD). Both treatment goals and prognosis may change with passing time. In order to discriminate between different moral issues faced by healthcare professionals in different care settings, i.e., rehabilitation center and nursing homes, MD’s will be organized both in EIN and PIN settings. We will use the Nijmegen method of moral deliberation [53], which aims to structure the multidisciplinary team conferences mainly in the situations of prospective decision-making. This method allows access to the ethical dimensions of the case by posing a clear moral question at the beginning of the case deliberation. During the first study year, various moral dilemmas occurring in EIN and PIN centers will be explored. After that, MD’s primarily concerning EOLD will be analyzed. Taking scenario’s registered in previous cohort studies into account, we aim to include five moral case deliberations or less until saturation is reached [11, 31, 41]. Only the multidisciplinary team involved in PDOC care will participate in MD’s.

#### Quality of outcomes and impact on patients and families

Evaluation of quality of outcomes in PDOC will be performed using both quantitative and qualitative data. DOCTOR uses various quantitative measures, such as the CRS-R for level of consciousness determination [54], Disability Rating Scale for functional level [55] and EuroQuol-5D, EQ-5D, a visual analogue scale [56], and QOLIBRI for quality of life assessment [57].

In TOPDOC the focus primarily lies on the views and perspectives of PDOC patients’ families and of the patients themselves who regain consciousness, regarding the outcomes of the rehabilitation trajectory. Patients and families will be first approached via telephone 2 years post-injury, in order to fill a comprehensive questionnaire on their personal views on the patient’s current situation. When a patient is unable to participate himself, only the views of family members will be inquired.

An in-depth qualitative interview will be held with the experiential expert regarding the quality of outcomes of rehabilitation (EIN and PIN). An example of the questions to be asked is: *“what does this outcome mean to you”?* These interviews will be performed by a trained researcher in a maximum of 10 patients selected on the basis of purposeful sampling. The views of treating EIN and PIN physicians on the outcomes of rehabilitation in PDOC will be explored during the interviews regarding treatment decisions and EOLD.

#### Dying in PDOC

DOCTOR will register mortality and cause of death in PDOC [51]. A distinction will be made between different causes and EOLD that precede death of a PDOC patient i.e.: death from comorbidity/complication despite treatment, death from comorbidity or complication after a non-treatment decision (withholding or withdrawing treatment) and death after withdrawal of CANH, as identified in previous studies [11, 19, 31, 41]. Based on current observations in DOCTOR, the expected number of deaths in the research population during the study period of 4 years is approximately 10. The perceptions of healthcare professionals and families of PDOC patients on the dying process following EOLD will be analyzed in TOPDOC.

A comprehensive questionnaire will be filled in by treating physicians within 2 weeks after death in all patients dying within 2 years post-injury. Information from the medical records regarding: (1) EOLD preceding death, (2) possible signs of discomfort in last 3 weeks of life, e.g., pain, shortness of breath etc., (3) palliative care e.g., use of medication like morphine and midazolam, (4) duration of dying process (5) cause of death and (6) complications occurring in the last 3 weeks of life will be obtained through the treating physician. Physicians and nurses involved in the terminal care for these 10 patients will be invited to participate in semi-structured in-depth interviews concerning abovementioned aspects within 2 months after the death of a PDOC patient.

Semi-structured interviews will also be conducted with these patient’s family members, provided that they give informed consent. Interviews will focus on the expectations and experiences of family members during the process of dying, their views regarding the preceding EOLD process and the guidance and supportive care provided by healthcare professionals to them. Example of questions to be asked are: “*How would you describe your loved one’s final days?”* “*Are there things you feel should have or could have been done in a better or different way?”*

Interviews will be conducted within 2 months after death and will be performed by a trained researcher with experience in PDOC care, but not involved in the terminal care of the patient.

#### Impact of providing care to PDOC patients and their families

The impact of providing care to PDOC patients and their families on healthcare professionals will be studied by means of direct observation of working environment at location and using focus-group methodology [58]. Focus group methodology is a qualitative approach to gain in-depth understanding of this issue. Physicians and nurses involved in care of PDOC patients, both in EIN and PIN settings, will be interviewed once in homogeneous groups of max 4–6 per discipline during focus group discussions.

Existing literature and observations will lead to a framework including specific questions concerning healthcare professionals’ experiences of providing care to PDOC patients and their families [50]. Focus group discussions will also explore the factors that may act as facilitators or barriers. Moreover, the needs of health-care professionals will be explored that might be helpful in doing their job. Following focus-group discussions, individual interviews will be carried out, only if necessary and until saturation is reached.

### Data acquisition timeline

Schematic representation of data acquisition is presented in Table 2.

**Table 2:** Data acquisition timepoints per study theme

Study theme	Timepoint of data collection	Patient OR Patients***’*** Family (time investment in minutes)	Treating physician (time investment in minutes)	Other participants (time investment in minutes)
**Treatment decisions (in a subset of minimum 6 cases)**	1 week after EIN admission	–	Questionnaire (20)	–
	3 months post-injury	–	Questionnaire (20) Qualitative interview (60)	–
	6 months post-injury	–	Questionnaire (20) Qualitative interview (60)	–
	1 year post injury	–	Questionnaire (20) Qualitative interview (60)	–
	2 year post-injury	–	Questionnaire (20) Qualitative interview (60)	–
**Ethical Dilemmas (in a subset of max 5 cases)**	During the course of study cohort	–	Moral deliberation (120)	Multidisciplinary team^a^ Moral deliberation (120)
**Dying in PDOC (in a subset of max 10 cases)**	2 months after death of the patient	Qualitative interview (90–120)	Questionnaire (20) Qualitative interview (60)	Nurses involved in terminal care Qualitative interview (90)
**Quality of outcomes (in a subset of max 10 cases)**	2 years post injury	Qualitative interview (90–120)	Questionnaire (10) Qualitative interview (30)	–
**Impact on health care professionals**	Independent of study cohort	–	Focus group discussion (120)	Nurses Focus group discussion (120)

^a^The Multidisciplinary team includes physiotherapist, occupational therapist, speech therapist, psychologist, social worker, nurses and physician

### Data management and analysis

Descriptive statistics will be performed to present the demographic features of the study population using SPSS. Comprehensive questionnaires will be formulated based on existing literature and previous research in cohort studies. The interviews will be conducted by experienced researchers with an affinity for PDOC care. Observation of moral deliberations and focus group discussions will be conducted by one researcher each. All interviews, moral deliberations and focus group discussions will be recorded with an audio equipment and then transcribed verbatim. In order to guarantee the methodological quality and transparent reporting on the complex themes involved in this study, we will carry out and report our findings according to the consolidated COREQ guidelines [59]. Data collection and analysis will alternate during the study and have a cyclical nature in order to gain an in-depth understanding of multiple research topics included in the study. The qualitative analysis will take place according to a thematic analysis [60]. The Atlas.ti programme will be used for this purpose. Multiple coders will independently encode the data and come to a single code tree based on consensus discussions.

## Discussion

After decades of suboptimal treatment and fragmented expertise for PDOC patients in the Netherlands, a newly established nationwide chain of PDOC care provides an opportunity to address existing knowledge gaps. Centralized expert-level rehabilitation aimed at recovery of consciousness is now available to all PDOC patients in a country with a tradition of open discussions about the quality- and end of life.

Two intertwined research projects, named TOPDOC and DOCTOR, will investigate several aspects of PDOC care in this context. Epidemiological facts and multiple quantitative outcomes in PDOC rehabilitation will be obtained in DOCTOR study [51]. Current study will increase our understanding of the complex process of EOLD and associated moral dilemmas in PDOC patients who have received optimal recovery-oriented therapy, an aspect which has not been studied extensively, especially in MCS patients. Moreover, provision of intensive neurorehabilitation for PDOC patients in a novel chain of care including both rehabilitation center and nursing home settings is a worldwide new and unique concept. In-depth analysis of what matters the most for the patients and their families when it comes to the outcomes of PDOC rehabilitation has not been studied in these settings. The impact of providing care to these patients and their loved ones on healthcare professionals working in these two different settings during first 2 years post-injury is an unexplored area of research.

TOPDOC also has its limitations. First, the study population is relatively small as a result of the low prevalence of PDOC, particularly in The Netherlands [2, 19, 41, 61]. The low prevalence in UWS has previously been linked to clinical decision-making and EOLD in both acute and chronic care settings [19, 61]. This study, however, will provide insight in both the process and the factors associated with decision-making in both UWS and MCS patients receiving specialized rehabilitation in first 2 years post-injury, which is a critical period for both treatment goals and decision-making [11].

Second, due to its qualitative nature, current study involves potentially burdensome methods with a risk of drop-out or non-consent, particularly in family members of PDOC patients, who are reported to show a high levels psychological distress [47]. However, this approach also provides new experiential insights on family perspectives and the impact of PDOC, which is valuable to improve PDOC care for future patients and their families. Families may also find comfort in sharing their stories and being heard [62].

Third, data arising from the Dutch context may seem less generalizable to international researchers and practitioners. Nevertheless, recent UK and AAN guidelines advocate establishment of national PDOC data registries and agree on the importance of a minimal dataset required for longitudinal assessments in order to improve PDOC care [5, 24, 63]. Due to the centralized PDOC care with EIN in a single rehabilitation center followed by PIN in nursing homes, DOCTOR-TOPDOC project covers a substantial proportion of Dutch adult PDOC population participating in specialized early and prolonged neurorehabilitation during first 2 years post-injury.

In conclusion, TOPDOC will facilitate better scientific understanding of PDOC care, optimize existing care pathways for PDOC patients and provide support and assistance to their families and healthcare professionals.

## Data Availability

Not applicable.

## Abbreviations

CANH: Clinically assisted nutrition and hydration
CRS-R: Coma Recovery Scale-Revised
ECP: Elderly care physician
EIN: Early Intensive Neurorehabilitation
EOLD: End of life decision(s)
LST: Life sustaining treatment
MCS: Minimally Conscious State
MD: Moral deliberation
NTBI: Non-Traumatic Brain Injury
PDOC: Prolonged Disorders of Consciousness
PIN: Prolonged Intensive Neurorehabilitation
UWS: Unresponsive Wakefulness Syndrome
